# Prediagnostic adult body mass index change and esophageal adenocarcinoma survival

**DOI:** 10.1002/cam4.3015

**Published:** 2020-03-23

**Authors:** Elizabeth A. Loehrer, Edward L. Giovannucci, Rebecca A. Betensky, Andrea Shafer, David C. Christiani

**Affiliations:** ^1^ Department of Environmental Health Harvard T.H. Chan School of Public Health Boston MA USA; ^2^ Department of Nutrition Harvard T.H. Chan School of Public Health Boston MA USA; ^3^ Department of Epidemiology Harvard T.H. Chan School of Public Health Boston MA USA; ^4^ Department of Biostatistics Harvard T.H. Chan School of Public Health Boston MA USA; ^5^ Pulmonary and Critical Care Division Massachusetts General Hospital Boston MA USA; ^6^ Present address: Department of Epidemiology Erasmus University Medical Center Rotterdam ZH the Netherlands; ^7^ Present address: Department of Biostatistics NYU School of Global Public Health New York NY United States

**Keywords:** adenocarcinoma, body mass index, body weight changes, esophageal neoplasms, survival analysis

## Abstract

**Background:**

We examined whether body mass index (BMI) changes in adulthood, prior to disease onset, are associated with overall survival among esophageal adenocarcinoma patients.

**Methods:**

We included 285 histologically confirmed patients with a complete baseline BMI questionnaire. Using extended Cox regression models, we obtained adjusted hazard ratios (HRs) for the associations between overall survival and BMI at diagnosis, BMI 6 months before diagnosis, self‐reported average adult BMI, and ΔBMI (BMI 6 months before diagnosis minus average adult BMI), categorized into tertiles <0 kg/m^2^ (BMI loss), ≥0 and <1.25 kg/m^2^ (stable BMI), and ≥1.25 kg/m^2^ (BMI gain). We also assessed interaction between ΔBMI and average adult BMI (≥ kg/m^2^ versus <27.5 kg/m^2^) with overall survival.

**Results:**

Body mass index at diagnosis >25 and <35 kg/m^2^ was associated with better overall survival. Compared to patients with stable BMI in adulthood, patients who gained BMI throughout adulthood had 1.68 times the all‐cause hazard of death (95% CI: 1.17‐2.43; *P* < .01), independent of diagnosis BMI and percent weight loss 6 months before diagnosis. Compared to patients with average adult BMI < 27.5 who maintained stable adult BMI, patients with average adult BMI ≥ 27.5 kg/m^2^ who gained BMI had the worst survival (HR = 3.05; 95% CI 1.62‐5.72; *P* < .01).

**Conclusion:**

Body mass index gain in adulthood is associated with poor overall survival, and maintaining a normal body weight throughout adulthood is associated with the best overall survival among esophageal adenocarcinoma patients, independent of BMI at diagnosis.

## Graphical abstract

Body mass index (BMI) gain in adulthood before cancer onset is associated with poor overall survival among esophageal adenocarcinoma patients, independent of BMI at diagnosis. Despite some reports that higher BMI may be protective for survival, we found no survival benefit of adding body weight prior to diagnosis, and suspect previous findings are driven by reverse causation.

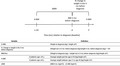

## 1 INTRODUCTION

Esophageal adenocarcinoma (EA) is the most common histological subtype of esophageal cancer in the western world, and fewer than 20% of patients survive 5 years.^1^ As with many cancer sites, obesity, measured as body mass index (BMI) > 30 kg/m^2^, is an established risk factor for EA,^2^, ^3^, ^4^, ^5^, ^6^, ^7^ but also as for many other cancer sites, the relationship of BMI and EA survival is less clear. To date, most studies of BMI as a prognostic marker in EA have focused on change in weight after esophagectomy or compared pretreatment weight to posttreatment weight. The results have been mixed, either indicating no association with BMI on survival time^8^, ^9^, ^10^, ^11^, ^12^, ^13^, ^14^, ^15^ or that patients with higher BMI have better overall survival.^15^, ^16^, ^17^, ^18^, ^19^ However, higher BMI at diagnosis (d‐BMI) and during treatment may in part indicate better overall health and lead to reverse causation of associations with overall survival in studies of postdiagnosis BMI.^20^, ^21^

Few studies have examined weight or weight change prior to disease onset as a prognostic factor in EA. BMI 1 year prior to diagnosis is not associated with overall survival.^22^, ^23^ Yet, one study showed that high BMI in early adulthood (age 18‐25) was associated with worse overall survival in EA.^22^ Additionally, substantial weight loss (>10% of body weight), after disease onset, in the months leading to diagnosis has been associated with poor overall survival in EA.^22^, ^24^

An estimated 80% of esophageal cancer patients report some weight loss in the 6 months prior to diagnosis,^25^, ^26^ but since high BMI is a strong risk factor for EA, most patients are nevertheless overweight or obese at the time of diagnosis despite the weight loss. Moreover, many patients have experienced in weight changes in adulthood separate from cancer‐related weight loss. Clinically, considering BMI from a single time point (eg, diagnosis) conflates patients who have had dynamic weight in adulthood prior to disease onset, patients with disease‐related weight loss, and patients who have had stable adult weight. To date, patients' adult weight changes prior to disease onset have not been considered as a prognostic factor. These weight changes may carry independent risks or benefits to disease outcomes and may help to elucidate the previously reported “obesity paradox” in EA.

Therefore, we examined whether changes in BMI in adulthood, prior to disease onset, were associated with overall survival among EA patients. Adult BMI (a‐BMI), d‐BMI, and weight change are three unique facets with potentially different associations with overall survival. Thus, to improve our understanding of the pattern of association with survival and BMI at different times, we first studied the association between overall survival and BMI at d‐BMI, BMI 6 months before diagnosis (BMI‐6mo), and self‐reported average a‐BMI. We then assessed the change from average a‐BMI to BMI‐6mo (ΔBMI) on overall survival time in EA patients. Finally, we hypothesized that a‐BMI changes may have different associations with survival depending on patients' starting BMI, so we tested whether the association with ΔBMI and overall survival in EA was modified by average a‐BMI.

## 2 METHODS

### 2.1 Study population

The Molecular Epidemiology of Esophageal Cancer^27^, ^28^ is an ongoing study of esophageal cancer patients from Massachusetts General Hospital (MGH), which began recruitment in January 1999. Recruited patients were >18 years of age with a histologically confirmed diagnosis who presented to the thoracic oncology or thoracic surgery units at MGH. The study protocol was approved by the institutional review boards at the Harvard T.H. Chan School of Public Health and MGH. Written informed consent was obtained from all patients prior to study participation. At the time of enrollment, a trained interviewer obtains patients' demographic and lifestyle information through a questionnaire. Since September 2004, the patient questionnaire included questions on weight throughout adulthood. The present study was restricted to histologically confirmed EA patients diagnosed between 9/1/2004 and 10/31/2016, who received the updated questionnaire. We excluded patients who were recruited at the time of cancer recurrence or cancer remission, who had a concurrent cancer, who only presented to MGH for a second opinion, or who were diagnosed with stage 0 disease. Of the 407 EA patients who met these criteria, 291 (71.5%) had complete information on weight throughout adulthood and were included in analyses (Figure S1). Among patients included in the study, median time between diagnosis and enrollment in the study was 4.8 weeks (IQR 2.1‐14.6 weeks). We determined patients' diagnosis date, clinical stage, and treatment regimen from clinical records.

### 2.2 Body mass index measurements

**FIGURE 1:**
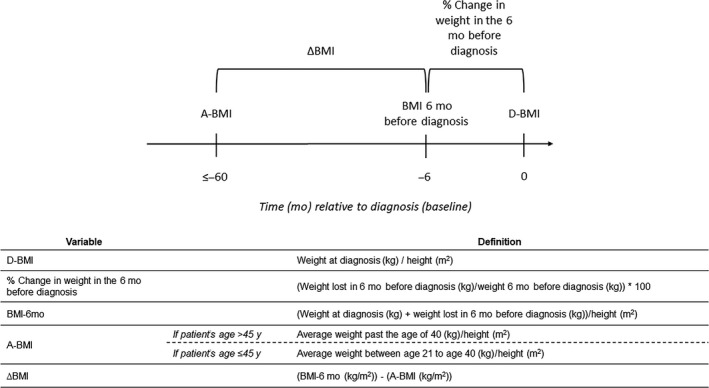
Schematic of exposure variables and their represented time (mo) relative to diagnosis (baseline)

During their questionnaire, patients reported their height, their average weight between age 18 and 21 years, their average weight between age 21 and 40 years, their average weight past the age of 40 years, and their weight loss in the 6 months before diagnosis, and their weight at time of diagnosis. Weight loss 6 months before diagnosis was assumed to be 0 for patients who did not answer that question but completed all other questions about BMI (N = 19). BMI was calculated as weight (kg) divided by height squared (m^2^). d‐BMI was based on self‐reported weight at diagnosis. BMI‐6mo was based on self‐reported weight at diagnosis plus self‐reported weight lost in the 6 months prior to diagnosis. For patients who were 45 years or older at time of diagnosis, a‐BMI was based on their self‐reported average weight past the age of 40 years. For patients younger than 45 years old at their time of diagnosis, a‐BMI was based on their self‐reported average weight between 21 and 40 years (Figure 1). Thus, a‐BMI represents average BMI over a period of at least 5 years prior to diagnosis, to capture the association of a‐BMI with overall survival, excluding weight loss that may occur closer to diagnosis, which is often related to the cancer. Once calculated, d‐BMI, BMI‐6mo, and a‐BMI were initially categorized into five groups: BMI < 18.5 kg/m^2^, 18.5 ≤ BMI < 25 kg/m^2^, 25 ≤ BMI < 30 kg/m^2^, 30 ≤ BMI < 35 kg/m^2^, and BMI ≥ 35 kg/m^2^. To minimize the capture of disease‐related weight loss that occurred close to diagnosis, ΔBMI was defined as the difference between a‐BMI and BMI‐6mo (Figure 1). We categorized ΔBMI into tertiles. The 33rd percentile of ΔBMI was 0 kg/m^2^ and the 66th was 1.25 kg/m^2^. ΔBMI < 0 kg/m^2^ corresponded to any BMI loss; ΔBMI ≥ 0 and <1.25 kg/m^2^ corresponded to stable BMI, which we used as our reference; and ΔBMI ≥ 1.25 kg/m^2^ corresponds to BMI gain. Correlations between BMI measurements are presented in Table S1.

### 2.3 Overall survival time

Overall survival time was defined as the time from date of pathology‐confirmed diagnosis until date of death (all‐cause mortality) or censored at date last known to be alive. Data on outcome measures were collected from clinical records and hospital cancer registries as well as through public death records, and are checked and updated approximately once per year. The last vital status update was conducted in August 2018 before this analysis.

### 2.4 Covariates

We modeled crude cigarette smoking history (never, former, and current) as an ordinal variable. Cancer stage at diagnosis was defined by clinical TNM categories based on American Joint Committee on Cancer (AJCC) 8th edition (Table S2), but due to the small number of patients in each stage, for analysis we categorized patients has lymph node negative, lymph node positive, or metastatic disease. Diagnosis date was considered date of pathology‐confirmed cancer. Treatment regimen was modeled as a series of binary variables: chemotherapy (yes/no), radiation (yes/no), and surgery (yes/no). Surgery was modeled as a time‐dependent covariate for two reasons. First, time between diagnosis and surgery is related to prognosis. Patients with early stage tumors receive esophagectomies as their definitive treatment, whereas locally advanced patients will receive esophagectomies pending their response to chemotherapy and/or radiation treatment. Second, the successful completion of the procedure is the most beneficial treatment for EA patient prognosis. Time of chemotherapy and radiation initiation was not collected, so we could not model chemotherapy or radiation as time‐dependent covariates. However, most patients who received chemotherapy and radiation did so prior to surgery or as definitive or palliative treatment (Table S3). We also adjusted for year of diagnosis to account for slight modifications to treatment protocols throughout the study period.

### 2.5 Statistical models

Predictors of d‐BMI and percent loss in body weight 6 months before diagnosis were tested using linear regression models. We used Kaplan‐Meier plots to visualize survival time distributions. Differences in survival curves between groups were formally tested using logrank tests. To estimate hazard ratios (HRs) of d‐BMI, BMI‐6mo, a‐BMI, and ΔBMI categories on overall survival, we used extended Cox regression models, adjusting for sex, age at diagnosis, smoking history, year of diagnosis, and treatment modality (chemotherapy, radiation, and/or surgery) with surgery modeled as a time‐dependent covariate. We also stratified baseline hazard by clinical stage lymph node status at diagnosis because proportional hazards did not hold across stage groups. For the association with ΔBMI categories, we ran a second model additionally adjusting for d‐BMI in addition to the aforementioned variables, and a third model additionally adjusted for percent body weight loss in the 6 months before diagnosis. To assess potential effect modification, we performed an analysis testing for interaction between ΔBMI and a‐BMI (modeled dichotomously as a‐BMI ≥ 27.5 versus a‐BMI < 27.5). All analyses were performed in SAS 9.4(SAS Institute Inc). *P*‐values were considered significant at a two‐sided alpha‐level of .05.

## 3 RESULTS

Of the 407 eligible patients, 291 EA patients (71.5%) had complete BMI information and were included in the analyses (Table 1). Due to the small number of patients with BMI < 18.5 kg/m^2^ (N = 6), we excluded those patients from the main analyses. While the small sample size precludes conclusion about associations in this group, we provided the same analyses with these six patients included in the supplemental material should readers wish to glean the pattern of association between body mass variables and overall survival for the underweight EA patients. Patients with and without complete BMI information did not differ in terms of overall survival (HR 1.13, 95% confidence interval [CI] 0.86‐1.49, *P* = .38, Figure S2). No patients reported weight gain in the 6 months prior to diagnosis. About 215 (73.9%) of patients reported some weight loss in the 6 months prior to diagnosis (median 6.1% loss in body weight [interquartile range 0%‐12.2%]). About 203 patients (69.8%) died during follow‐up. The median (Kaplan‐Meier) survival time for all patients was 30.7 months (95% CI 26.0‐36.1 months).

**TABLE 1:** Demographics of the study population

	With adult BMI available (N = 291)	Without adult BMI available (N = 116)
Male	260 (89.4%)	95 (81.9%)
Age	63.1 ± 10.3	64.1 ± 10.2
Race		
White	279 (95.9%)	92 (79.3%)
Black	1 (0.3%)	
Hispanic	4 (1.4%)	1 (0.9%)
Asian	1 (0.3%)	2 (1.7%)
Native American	4 (1.4%)	2 (1.7%)
Smoking status		
Never	78 (26.8%)	17 (14.7%)
Former	184 (63.2%)	64 (55.2%)
Current	29 (10.0%)	35 (30.2%)
Stage		
Lymph node positive	93 (32.0%)	49 (42.2%)
Lymph node negative	127 (43.6%)	56 (48.3%)
Distant metastases	71 (24.4%)	11 (9.5%)
Treatment^a^		
Surgery	191 (65.6%)	96 (82.8%)
Radiation	206 (70.8%)	82 (70.7%)
Chemotherapy	240 (82.5%)	86 (74.1%)
Diagnosis BMI (kg/m^2^)		
<18	6 (2.1%)	1 (0.9%)
≥18.5 and <25	81 (27.8%)	30 (25.9%)
≥25 and <30	129 (44.3%)	48 (41.4%)
≥30 and <35	58 (19.9%)	21 (18.1%)
≥35	17 (5.8%)	15 (12.9%)
Average adult BMI (kg/m^2^)		
<18	1 (0.3%)	
≥18.5 and<25	38 (13.1%)	
≥25 and<30	158 (54.3%)	
≥30 and <35	69 (23.7%)	
≥35	25 (8.6%)	
ΔBMI (kg/m^2^)		
<0	79 (27.2%)	
≥0 and <1.25	113 (38.8%)	
≥1.25	99 (34.0%)	
Deaths	203 (69.8%)	68 (58.6%)

Note

Values presented are mean ± standard deviation or absolute number (population %).

Abbreviation: BMI, body mass index.

^a^ Treatment categories are not mutually exclusive.

### 3.1 BMI at single time‐points

Each percent point of body weight lost in the 6 months before diagnosis was associated with a 0.18 kg/m^2^ lower BMI at the time of diagnosis (Table S4). Patients who gained BMI in adulthood (∆BMI ≥ 1.25 kg/m^2^) (N = 99) had on average 3.59 kg/m^2^ higher d‐BMI compared to those with stable weight throughout adulthood (N = 113) (*P* < .01, Table S4).

Overall survival time did not differ by d‐BMI in the unadjusted models (Figure S3: logrank *P*‐value = .08; Table 2). In the adjusted model, d‐BMI was associated with overall survival (global Wald *P*‐value = .01, Table 2; Table S6), with lowest hazard of death among patients with d‐BMI ≥ 25 and <35 kg/m^2^. As a sensitivity analysis to check for potential selection bias, we tested the association between overall survival and d‐BMI in the full cohort (Figure S1: N = 407), and found a similar pattern of association, though the associations strengthened in the larger cohort (Table S7).

**TABLE 2:** Average adult BMI, BMI 6 mo before diagnosis, BMI at the time of diagnosis, and overall survival among EA patients (N = 285)

	Univariable models					Multivariable models			
	N events/patients	Unadjusted HR	95% CI	*P*‐value	Global *P*‐value^a^	Adjusted HR^a^	95% CI	*P*‐value	Global *P*‐value^b^
a‐BMI categories (kg/m^2^)									
≥18.5 and <25	21/34	Ref			.52	Ref			.68
≥25 and <30	110/157	1.34	0.84; 2.14	.22		1.15	0.71; 1.85	.58	
≥30 and <35	48/69	1.47	0.88; 2.46	.14		1.30	0.76; 2.23	.35	
≥35	20/25	1.42	0.77; 2.63	.26		1.42	0.74; 2.69	.29	
BMI‐6mo categories (kg/m^2^)									
≥18.5 and <25	21/30	Ref			.56	Ref			.18
≥25 and <30	87/131	1.05	0.65; 1.69	.85		0.72	0.44; 1.20	.72	
≥30 and <35	63/84	1.29	0.79; 2.12	.31		0.97	0.58; 1.62	.97	
≥35	28/40	1.21	0.69; 2.14	.51		1.09	0.60; 1.98	.77	
d‐BMI categories (kg/m^2^)									
≥18.5 and <25	61/81	Ref			.08	Ref			<.01
≥25 and <30	88/129	0.77	0.55; 1.06	.11		0.60	0.42; 0.84	<.01	
≥30 and <35	36/58	0.63	0.42; 0.95	.03		0.62	0.41; 0.96	.03	
≥35	14/17	1.14	0.64; 2.04	.66		1.22	0.66; 2.42	.53	

Abbreviation: BMI, body mass index.

^a^ Adjusted for sex, age at diagnosis, smoking status, treatment and year of diagnosis. The model's baseline hazard was stratified by lymph node status, and surgery was a time‐dependent covariate.

^b^ Global Wald test with 3 degrees of freedom. Due to small numbers, patients with BMI < 18.5kg/m^2^ (N = 6) were excluded from these analyses.

We found no association between categories of BMI‐6mo (Figure S4) or a‐BMI (Figure S5) and overall survival time by logrank test, nor did we find a difference in unadjusted or adjusted HRs for categories of BMI‐6mo or a‐BMI (Table 2; Table S6).

### 3.2 Percent body weight lost in 6 months before diagnosis

Current smokers lost on average 4.79% more body weight in the 6 months prior to diagnosis than never smokers (Table S5). Patients who lost BMI in adulthood (∆BMI < 0 kg/m^2^; N = 79) on average had 1.93% less loss in body weight in the 6 months before diagnosis (*P* = .07, Table S5). For each percentage point increase in body weight lost in the 6 months prior to diagnosis, the all‐cause hazard of death for EA patients increased 1.03 times (95% CI 1.01‐1.05; *P* < .001; data not in table).

### 3.3 ∆BMI: adult BMI change up to 6 months before diagnosis

In the unadjusted association of a‐BMI change (∆BMI) and overall survival, we found no evidence of difference between the survival times (Figure S6). Compared to patients with stable a‐BMI (N = 111), patients who lost BMI in adulthood (N = 75) had 1.16 times the all‐cause hazard of death (95% CI 0.82‐1.63, *P* = .41), and patients who gained BMI in adulthood (N = 99) had 1.32 times the all‐cause hazard of death (95% CI 0.95‐1.83; *P* = .10) (data not in table). When adjusted for confounders and predictors of overall survival, we found patients who gained BMI in adulthood had 1.53 times higher all‐cause hazard of death compared to patients who had stable a‐BMI (Table 3, Model 1). The association became more pronounced (HR = 1.68, 95% CI 1.17‐2.42) when additionally adjusting the model for d‐BMI and percent body weight loss 6 months prior to diagnosis (Table 3, Model 3; Table S8). Moreover, both d‐BMI and percent loss of weight in 6 months prior to diagnosis remain significantly associated with all‐cause hazard of death in the fully adjusted model (Table 3, Model 3; Table S8). In these analyses, we did not find evidence that patients who lost BMI in adulthood prior to disease onset (ΔBMI < 0) had a different all‐cause hazard of death compared to patients who had stable a‐BMI.

**TABLE 3:** ΔBMI between average adult weight and weight 6 months prior to diagnosis and overall survival among EA patients (N = 285)

	N events/patients	Model 1				Model 2				Model 3			
		HR^a^, ^b^, ^a^	95% CI	*P*‐value	Global *P*‐value	HR^a^, ^b^, ^a^	95% CI	*P*‐value	Global *P*‐value	HR^c^, ^b^	95% CI	*P*‐value	Global *P*‐value
∆BMI categories (kg/m^2^)													
Stable: >0 and ≤1.25	72/111	Ref			.02^d^	Ref			<.01^d^	Ref			.01^d^
BMI loss: <0	54/75	1.03	0.72; 1.48	.87		0.98	0.68; 1.42	.92		1.04	0.72; 1.51	.83	
BMI gain: ≥1.25	73/99	1.53	1.09; 2.14	.01		1.76	1.23; 2.54	<.01		1.68	1.17; 2.43	<.01	
d‐BMI (kg/m^2^)													
≥18.5≥ and <25	61/81					Ref			<.01^e^	Ref			<.01^e^
≥25 and <30	88/129					0.51	0.36; 0.73	<.01		0.54	0.38; 0.79	<.01	
≥30 and <35	36/58					0.49	0.31; 0.77	<.01		0.54	0.34; 0.86	<.01	
≥35	14/17					0.83	0.43; 1.60	.59		0.95	0.49; 1.85	.89	
% body weight loss 6‐mo prior to diagnosis^e^										1.02	1.00; 1.04	.03	

Abbreviation: BMI, body mass index.

^a^ Model 1: adjusted for sex, age at diagnosis, smoking status, treatment, and year of diagnosis. The model's baseline hazard was stratified by lymph node status, and surgery was coded as a time‐dependent covariate.

^b^ Model 2: additionally adjusted for d‐BMI.

^c^ Model 3: additionally adjusted for percent body weight loss 6 mo before diagnosis.

^d^ Global Wald test with 2 *df*.

^e^ Global Wald test with 3 *df*. Due to the small numbers, patients with BMI < 18.5 kg/m^2^ (N = 6) were excluded from these analyses.

### 3.4 Interaction between average a‐BMI and ∆BMI

We examined whether the association between adult weight change (∆BMI) and overall survival differed depending on patients' average a‐BMI. Due to the small number of patients who had a‐BMI < 25.0 kg/m^2^ (N = 34), we dichotomized a‐BMI at the half‐way point of the overweight category (a‐BMI < 27.5 kg/m^2^ versus a‐BMI ≥ 27.5 kg/m^2^) to represent adult lean and adult overweight/obese patients, recognizing that patients who had an average a‐BMI of 25‐27.6 did have some excess mass (likely excess adiposity), but also noting that they were still at the lower end of the BMI range in this population. Adult lean patients who had stable BMI in adulthood (reference: a‐BMI < 27.5 kg/m^2^ and ∆BMI 0‐1.25 kg/m^2^; N = 53) had the best survival of all groups, whereas adult overweight/obese patients who gained BMI in adulthood (N = 57) had the worst survival, with three times the all‐cause hazard of death (Table 4). Compared to adult lean patients with stable BMI in adulthood, BMI loss in adulthood was associated with approximately 1.5 times hazard of death among both adult lean patients (N = 19) and adult obese patients (N = 56), though neither association was statistically significant (Table 4). However, when comparing only among adult obese patients (a‐BMI ≥ 27.5 kg/m^2^), patients who lost BMI in adulthood (N = 56) had 0.69 times the all‐cause hazard of death compared against patients with stable BMI in adulthood (N = 58), though it was not statistically significant (95% CI 0.42, 1.11; *P* = .13). Including the six patients with d‐BMI < 18.5 kg/m^2^ in this analysis yielded similar results (Table S9).

**TABLE 4:** Interaction between ΔBMI and average adult BMI and overall survival among EA patients (N = 285)

∆BMI categories (kg/m^2^)		N events/patients	HR^a^, ^b^, ^a^	95% CI	*P*‐value	Interaction *P*‐value^a^, ^b^, ^a^
Stable: ≥0 and <1.25	a‐BMI < 27.5	29/53	Ref			.10
BMI loss: <0		17/19	1.48	0.80; 2.77	.21	
BMI gain: ≥1.25		31/42	2.59	1.44; 4.66	<.01	
Stable: ≥0 and <1.25	a‐BMI ≥ 27.5	43/58	2.24	1.24; 4.04	<.01	
BMI loss: <0		37/56	1.54	0.90; 2.62	.11	
BMI gain: ≥1.25		42/57	3.05	1.62; 5.72	<.01	

Abbreviation: BMI, body mass index.

^a^ Adjusted for sex, age at diagnosis, smoking status, treatment, year of diagnosis, diagnosis BMI, percent body weight lost within the 6 mo before diagnosis. The baseline hazard was stratified by lymph node status, and surgery was coded as a time‐dependent covariate.

^b^ Wald test *P*‐value for interaction term. Due to small numbers, patients with BMI < 18.5 kg/m^2^ (N = 6) were excluded from these analyses.

## 4 DISCUSSION

To our knowledge, this is the first study to assess the association of adult weight change prior to cancer onset with overall survival in EA patients. Adults who gained BMI throughout adulthood (∆BMI ≥ 1.25 kg/m^2^) had worse overall survival compared to patients who maintained stable BMI throughout adulthood, independent of BMI at time of diagnosis and percent weight loss in the 6 months prior to diagnosis. BMI loss in adulthood prior to disease onset (∆BMI < 0 kg/m^2^) was not associated with poorer survival compared to patients who maintained stable BMI throughout adulthood. These results suggest no benefit of accumulating or maintaining fat stores prior to the disease process; if additional fat stores causally improve survival, gaining additional fat in adulthood should improve survival, particularly among lean patients.

Consistent with previous studies that examined only d‐BMI and weight loss leading up to diagnosis, we found that patients with BMI ≥ 25.0 and <35 kg/m^2^ at the time of diagnosis (d‐BMI) had an decreased hazard of all‐cause mortality compared to patients with d‐BMI 18.5‐25 kg/m^2^ ^15^, ^16^, ^17^, ^18^, ^19^ and weight loss in the 6 months before diagnosis^11^, ^22^, ^24^, ^29^ was also associated with an increased hazard of all‐cause mortality. Accounting for ∆BMI did not attenuate these associations. Yet, because weight loss only in the 6 months prior to diagnosis, but not previous weight loss, was associated with poorer survival, the remaining observed association between d‐BMI < 25 kg/m^2^ and overall survival is likely capturing overall poor health status and complex unaccounted clinical factors (reverse causation and residual confounding).

Still, early life obesity and weight gain may biologically influence esophageal adenocarcinoma prognosis differently than BMI at the time of diagnosis. Given the invasive and aggressive treatment of esophageal adenocarcinoma, we cannot rule out that higher BMI at the time of diagnosis has a biological survival advantage which may contrast from the effects of prediagnosis BMI.

Notably, lean adult patients who had stable BMI throughout adulthood had the lowest hazard rate of all groups. Among both a‐BMI < 27.5 kg/m^2^ and a‐BMI ≥ 27.5 kg/m^2^, we observed that BMI gain (∆BMI ≥ 1.25 kg/m^2^) in adulthood was associated with increased hazard of death compared to patients with stable BMI. Though we cannot determine the underlying causal mechanisms of the association, one hypothesis is that BMI gain in adulthood (at all weights) confers a risk of a more aggressive tumor subtype, leading to poorer survival outcomes postdiagnosis. Among only patients with a‐BMI ≥ 27.5 kg/m^2^, those who lost BMI in adulthood had a seemingly better survival compared to patients with stable BMI in adulthood, though the difference was not statistically significant. One cautious interpretation of this observation is that BMI loss among patients with a‐BMI ≥ 27.5 kg/m^2^ may represent a mix of patients with residual disease‐related weight loss (deleterious) and patients with intentional weight loss (beneficial). Additionally, patients with a‐BMI ≥ 27.5 kg/m^2^ who had stable BMI or BMI gain in adulthood may be more likely than other subgroups to experience continued acid reflux, which could contribute to cancer progression in these patients. Ultimately, our findings highlight the insufficiency of using d‐BMI alone to conclude that higher weight at diagnosis reflects a benefit for patient prognosis.

We could not take into account body composition in this study. We used BMI as a proxy for adiposity, but BMI is correlated less with adiposity in the elderly or in cancer patients, who both have tendencies for sarcopenia (low lean muscle mass).^30^, ^31^ Moreover, the decline of muscle mass with aging seems to be steeper among men than women,^32^, ^33^, ^34^, ^35^ though this may be mitigated by physical activitity.^35^ Sarcopenia is associated with both cachexia, a cancer‐wasting syndrome, and with poor survival in cancer.^26^, ^36^, ^37^ Accounting for the proportion of adiposity and lean muscle mass in patients is important to assess the driving mechanisms in the association with mass and survival in EA. However, studies of sarcopenia and survival in esophageal cancer to date have also relied largely on postdiagnosis measurements and changes, so these baseline measurements still do not account for prediagnosis change in body composition. Our results should add caution by emphasizing that metrics of body mass at the time of diagnosis, no matter how sophisticated, may be misleading if we do not account for the path of how the person attained their weight. Due to the primary study on the prognostic value of body composition after diagnosis, it remains unclear whether body composition in a person's adult life prior to disease onset effects cancer prognosis through the same mechanism as observed postdiagnosis. Studies of prediagnostic body composition with adult weight change in EA may prove particularly useful in elucidating metabolic mechanisms influencing cancer outcomes and identifying patients at risk of cachexia earlier in their clinical course.

Although nearly 30% of our population was missing information on a‐BMI, we found no difference in overall survival and found similar associations between d‐BMI and overall survival for patients who did and did not report earlier life BMI measures, lowering the concern for selection bias. We relied on self‐reported prediagnostic average adult weight and diagnostic weight. Though patients completed the questionnaire close to the time of diagnosis (median time after diagnosis 4.8 weeks), there may have been increased recall error the longer after diagnosis patients completed the questionnaire. d‐BMI and reporting of weight loss in the 6 months prior to diagnosis are expected to be accurate.^38^, ^39^, ^40^ Studies have also shown that self‐reported weight from previous times in life is fairly accurate,^41^, ^42^, ^43^ though obese patients may have greater misclassification of their early life‐weight.^42^, ^43^, ^44^ Similarly, older patients had diluted average a‐BMI values, since the average a‐BMI value does not capture weight fluctuation in later adulthood. However, given EA's general clinical aggressiveness, age at diagnosis is not a strong predictor of overall survival, indicating this misclassification would have a weak impact on our association estimates. Additionally, we did not have information on cause‐specific mortality, but due to the aggressive pattern of EA, we infer that the vast majority of patients in our study died from their cancer.

To our knowledge, we are the first to investigate the prognostic value of prediagnostic adult weight changes, independent of disease‐related weight loss, in EA. We have a relatively large sample size of EA patients. In contrast to previous studies that used weight 1 year prior to diagnosis, we used average adult weight at least 5 years before diagnosis to reduce the possibility of disease‐related changes with that measure. Additionally, unlike many clinical studies, we have thorough demographic and lifestyle data collected systematically for the purposes of research.

Our findings indicate that BMI gain in adulthood is associated with poor overall survival, and maintaining a normal body weight throughout adulthood is associated with the best overall survival among EA patients. Changes in BMI around the time of diagnosis likely reflect the consequences of the disease.

## CONFLICT OF INTEREST

The authors of this manuscript report no conflicts of interest.

## AUTHOR CONTRIBUTION

EL, EG, RB, and DC conceived the study question and design. EL, EG, RB developed analysis plan. AS performed data collection. EL performed analyses. EL, EG, RB, and DC contributed to interpretation of the results. EL took the lead on writing the manuscript. All authors provided critical feedback and helped shape the research and manuscript. The work was conducted in the lab of DC, who also provided mentorship for the project.

## Supporting information

Fig S1

Fig S2

Fig S3

Fig S4

Fig S5

Fig S6

Table S1‐S9

## Data Availability

Availability of data and material. Our IRB consent procedures at the time this study was done do not allow individual level data sharing. We can share aggregate data that may be used in meta‐analyses or similar approaches. Please contact the corresponding author.
