# Supplementary figures and images for: Prediagnostic adult body mass index change and esophageal adenocarcinoma survival
Source: Cancer Med. 2020 Mar 23;9(10):3613–22. doi: 10.1002/cam4.3015 (PMC7221446; doi:10.1002/cam4.3015)

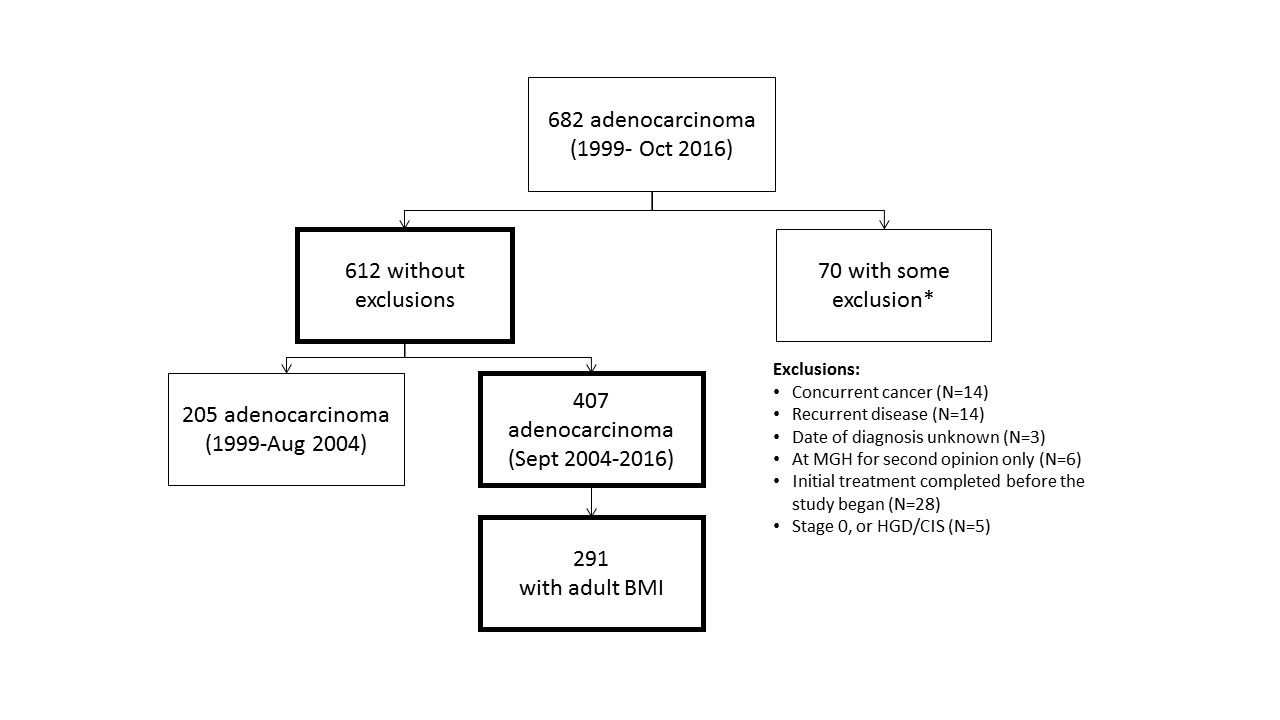

Supplement: Supplementary file 1 — Fig S1 [file CAM4-9-3613-s001.tif]

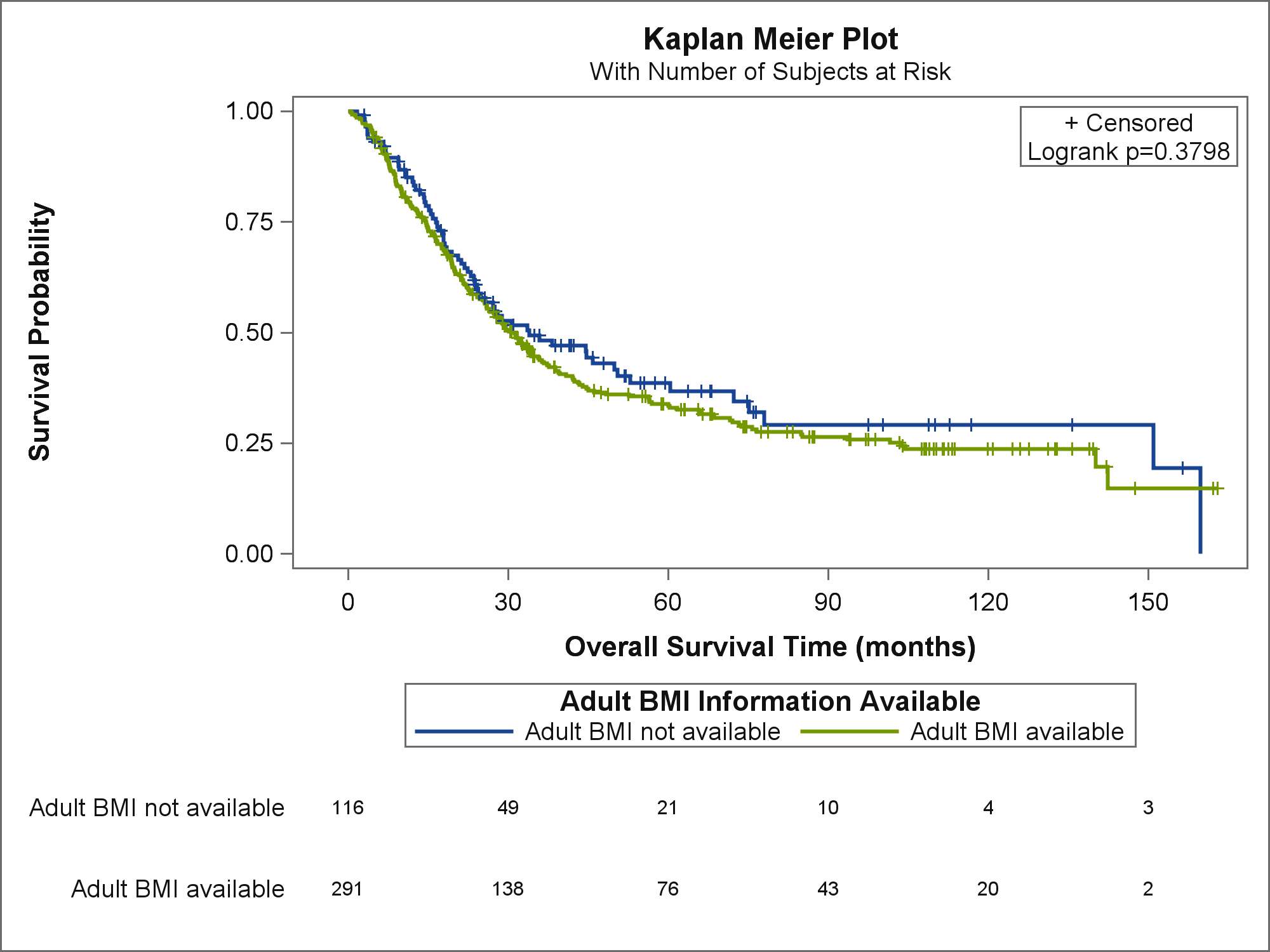

Supplement: Supplementary file 2 — Fig S2 [file CAM4-9-3613-s002.tif]

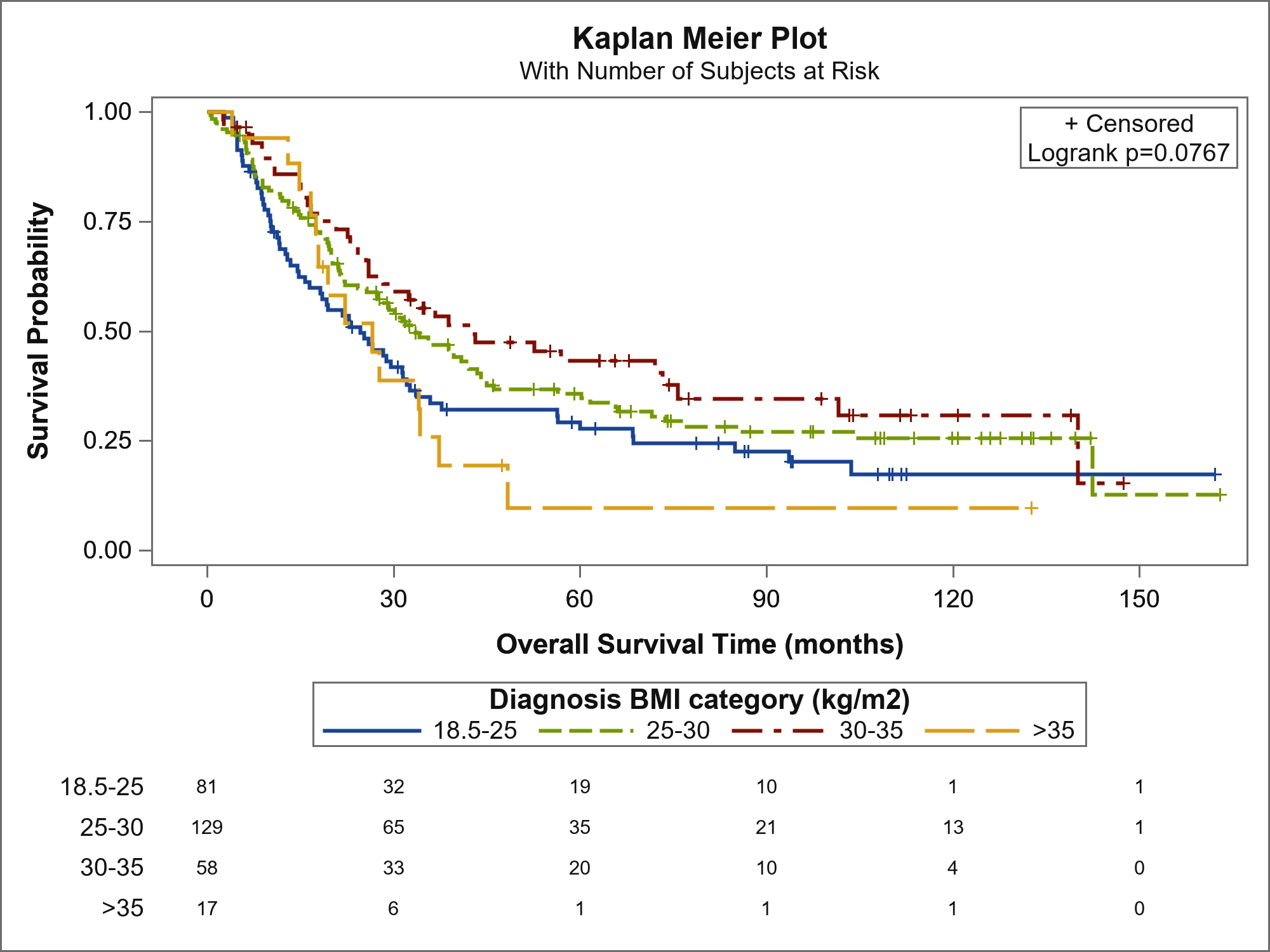

Supplement: Supplementary file 3 — Fig S3 [file CAM4-9-3613-s003.png]

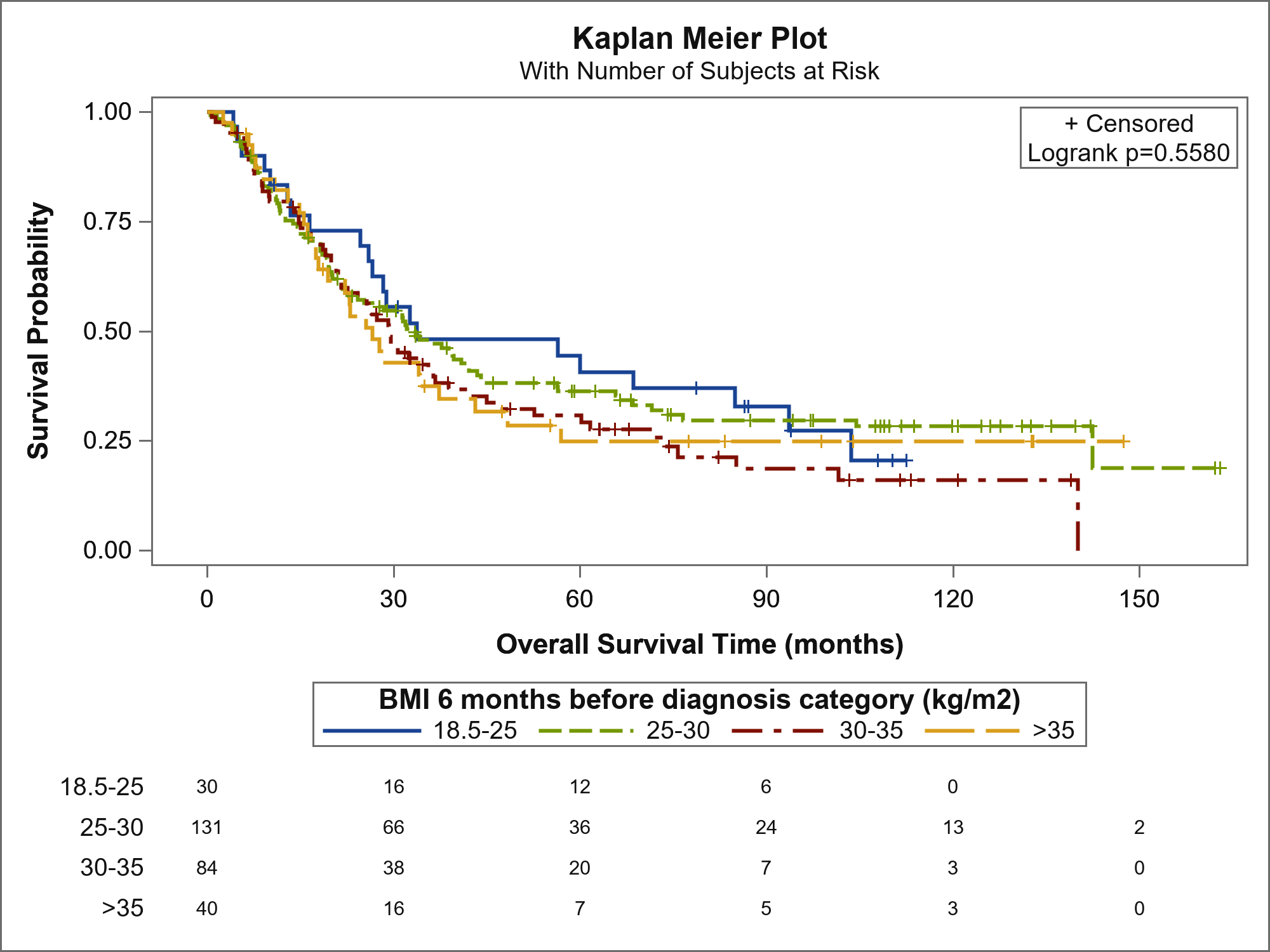

Supplement: Supplementary file 4 — Fig S4 [file CAM4-9-3613-s004.png]

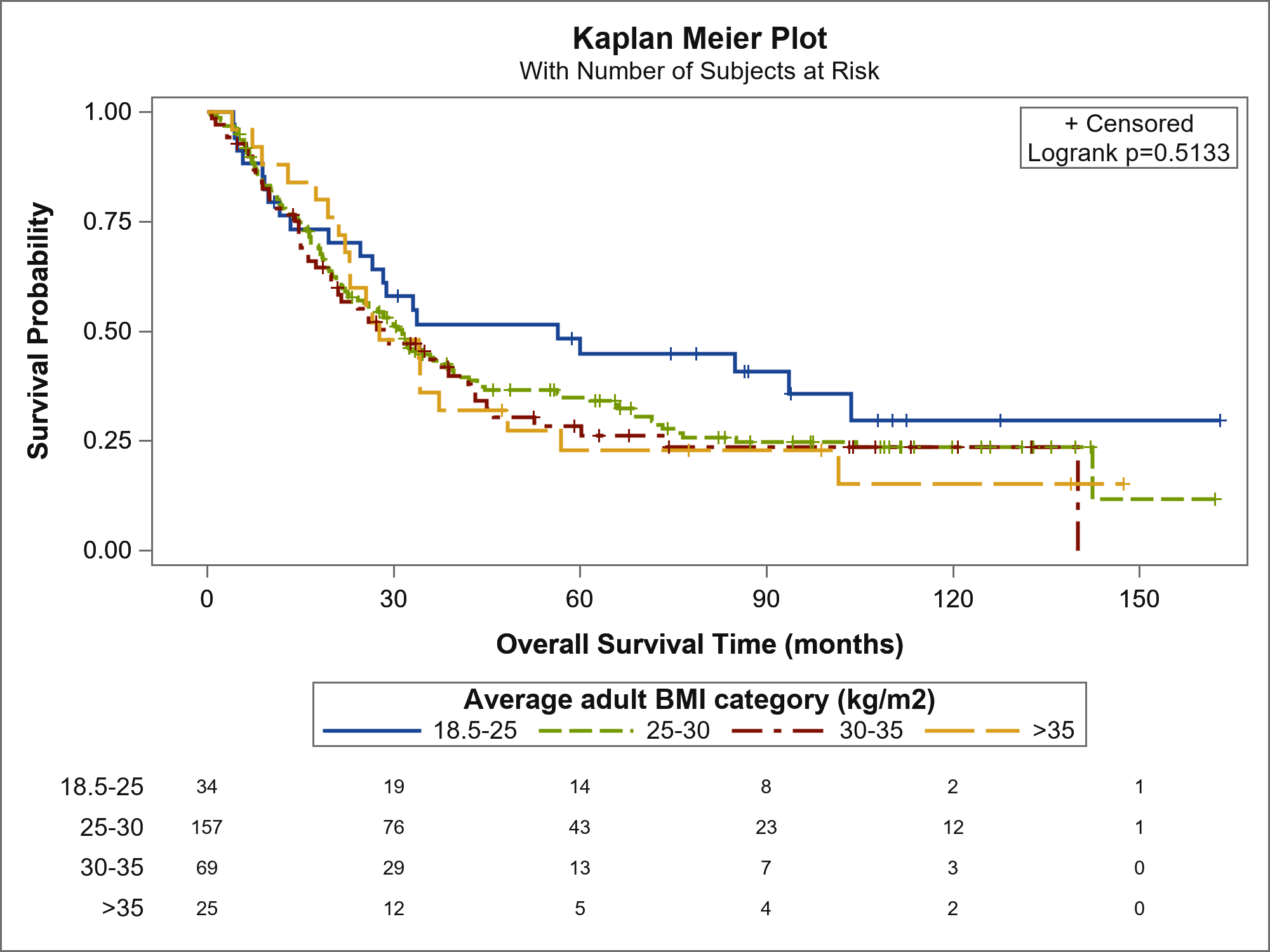

Supplement: Supplementary file 5 — Fig S5 [file CAM4-9-3613-s005.png]

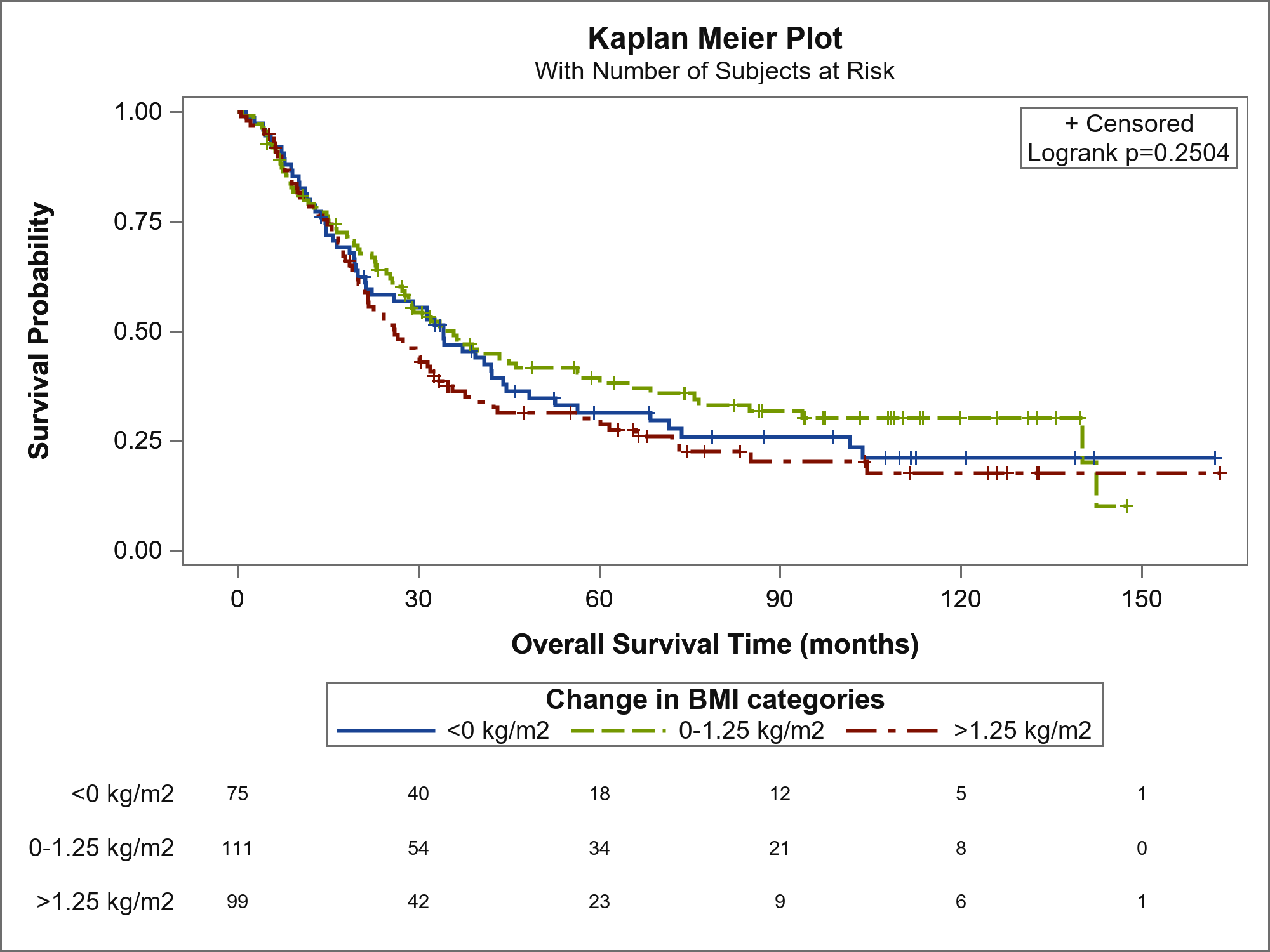

Supplement: Supplementary file 6 — Fig S6 [file CAM4-9-3613-s006.png]
