# Supplementary material for: Prediagnostic adult body mass index change and esophageal adenocarcinoma survival
Source: Cancer Med. 2020 Mar 23;9(10):3613–22. doi: 10.1002/cam4.3015 (PMC7221446; doi:10.1002/cam4.3015)
Supplement: Supplementary file 7 — Table S1‐S9 [file CAM4-9-3613-s007.docx]

Supplementary Figure Legends

**Supplementary Figure 1: Flow chart of the study population**

The flow chart describes the inclusion and exclusion criteria and how we arrived at the population for analyses.

**Supplementary Figure 2: Kaplan-Meier curve for overall survival: patients with and without complete BMI information**

**Supplementary Figure 3: Kaplan-Meier curve for overall survival by BMI at diagnosis category**

**Supplementary Figure 4: Kaplan-Meier curve for overall survival by BMI 6 months before diagnosis category**

**Supplementary Figure 5: Kaplan-Meier curve for overall survival by average adult BMI category**

**Supplementary Figure 6: Kaplan-Meier curve for overall survival by ∆BMI category**

| **Supplementary Table 1: Correlation between exposure measurements** | | | | |
| --- | --- | --- | --- | --- |
| **A-BMI** |  |  |  |  |
| 0.74** | **BMI-6mo** |  |  |  |
| 0.64** | 0.79** | **D-BMI** |  |  |
| 0.13* | 0.19** | 0.25** | **% bodyweight loss 6-months to diagnosis** |  |
| -0.04 | 0.36** | 0.36** | 0.07 | **∆BMI** |
|  |  |  |  |  |

Spearman correlation coefficients comparing each of the body mass index and weight exposure metrics. *Denotes p-value<0.05. **Denotes p-value<0.001.

| **Supplementary Table 2: Clinical Stage by AJCC 8^th^ edition TNM classification** | | | | |
| --- | --- | --- | --- | --- |
|  |  | **With adult BMI available (N=291)** |  | **Without adult BMI available (N=116)** |
| **Stage** |  |  |  |  |
| **I** |  | 35 (12.0%) |  | 22 (19.0%) |
| **IIA** |  | 9 (3.1 %) |  | 4 (3.5%) |
| **IIB** |  | 26 (8.9%) |  | 9 (7.8%) |
| **III** |  | 130 (44.7%) |  | 56 (48.3%) |
| **IVA** |  | 20 (6.9%) |  | 14 (12.1%) |
| **IVB** |  | 71 (24.4%) |  | 11 (9.5%) |

Values represent number (%) of patients in each category.

| **Supplementary Table 3: Treatment modality order of study participants (N=291)** | | |
| --- | --- | --- |
|  |  |  |
| Surgery alone |  | 41 (14.1%) |
| Chemotherapy alone |  | 35 (12.0%) |
| Radiation alone |  | 4 (1.4%) |
| Chemotherapy then surgery |  | 3 (1.0%) |
| Surgery then chemotherapy |  | 1 (0.3%) |
| Surgery then radiation |  | 1 (0.3%) |
| Chemotherapy and radiation |  | 56 (19.2%) |
| Trimodality (chemotherapy and radiation followed by surgical resection) |  | 145 (49.8%) |
| No Treatment |  | 5 (1.7%) |

Values represent number (%) of patients in each category.

| **Supplementary Table 4: Predictors of body mass index at diagnosis (d-BMI) (N=291)** | | | | | | | |
| --- | --- | --- | --- | --- | --- | --- | --- |
|  |  | | |  |  | | |
|  | **Univariable models** | | |  | **Multivariable model^†^** | | |
|  | **Change in d-BMI (kg/m^2^)** | **95% CI** | **p-value** |  | **Change in d-BMI (kg/m^2^)** | **95% CI** | **p-value** |
| **Percent body weight loss 6 months before diagnosis** | -0.18 | (-0.25; -0.11) | <0.01 |  | -0.19 | (-0.26; -0.12) | <0.01 |
|  |  |  |  |  |  |  |  |
| **∆BMI Categories (kg/m^2^)** |  |  |  |  |  |  |  |
| BMI loss: <0 | -0.06 | (-1.34; 1.22) | 0.93 |  | -0.50 | (-1.74; 0.73) | 0.43 |
| BMI gain: >1.25 | 3.59 | (2.39; 4.79) | <0.01 |  | 3.46 | (2.29; 4.63) | <0.01 |
| Stable: >0 and ≤1.25 | Ref |  |  |  | Ref |  |  |
|  |  |  |  |  |  |  |  |
| **Smoking Categories** |  |  |  |  |  |  |  |
| Current Smoker | -0.21 | (-2.23; 1.81) | 0.84 |  | 0.63 | (-1.22; 2.48) | 0.50 |
| Former Smoker | 1.08 | (-0.18; 2.33) | 0.09 |  | 0.63 | (-0.55; 1.82) | 0.29 |
| Never Smoker | Ref |  |  |  |  |  |  |
|  |  |  |  |  |  |  |  |
| **Age at diagnosis** | 0.00 | (-0.05; 0.06) | 0.95 |  | -0.02 | (-0.07; 0.03) | 0.35 |
|  |  |  |  |  |  |  |  |
| **Male sex** | -0.27 | (-2.04; 1.50) | 0.77 |  | -0.72 | (-2.31; 0.88) | 0.38 |

Values represent regression coefficient, 95% confidence interval and p-value for linear regression with d-BMI as the dependent variable. ^†^Multivariable model values are adjusted for all other variables in the table.

| **Supplementary Table 5: Predictors of percent body weight loss 6 months before diagnosis (N=291)** | | | | | | | |
| --- | --- | --- | --- | --- | --- | --- | --- |
|  |  | | |  |  | | |
|  | **Univariable models** | | |  | **Multivariable model**^†^ | | |
|  | **Difference in % body weight lost 6 months before diagnosis** | **95% CI** | **p-value** |  | **Difference in % body weight lost 6 months before diagnosis** | **95% CI** | **p-value** |
| **∆BMI Categories (kg/m^2^)** |  |  |  |  |  |  |  |
| BMI loss: <0 | -2.07 | (-4.20; 0.05) | 0.06 |  | -1.93 | (-4.03; 0.18) | 0.07 |
| BMI gain: >1.25 | -0.54 | (-2.53; 1.46) | 0.60 |  | -0.26 | (-2.27; 1.74) | 0.80 |
| Stable: >0 and ≤1.25 | Ref |  |  |  | Ref |  |  |
|  |  |  |  |  |  |  |  |
| **Smoking Categories** |  |  |  |  |  |  |  |
| Current Smoker | 4.85 | (1.74; 7.96) | <0.01 |  | 4.79 | (1.67; 7.91) | <0.01 |
| Former Smoker | -0.12 | (-2.05; 1.81) | 0.90 |  | -0.04 | (-2.08; 1.99) | 0.96 |
| Never Smoker | Ref |  |  |  |  |  |  |
|  |  |  |  |  |  |  |  |
| **Age at diagnosis** | -0.04 | (-0.13; 0.04) | 0.30 |  | -0.01 | (-0.10; 0.07) | 0.75 |
|  |  |  |  |  |  |  |  |
| **Male sex** | 0.71 | (-2.05; 3.48) | 0.61 |  | 0.48 | (-2.26; 3.22) | 0.73 |

Values represent regression coefficient, 95% confidence interval and p-value for linear regression with percent body weight lost 6 months before diagnosis as the dependent variable. Positive values represent a larger percentage of bodyweight lost (more weight loss prior to diagnosis), and negative values represent a lower percentage of bodyweight lost (less weight loss prior to diagnosis) ^†^Multivariable model values are adjusted for all other variables in the table.

| **Supplementary Table 6: Average adult BMI, BMI 6 months before diagnosis, BMI at the time of diagnosis, and overall survival among EA patients (N=291)** | | | | | | | | | | |
| --- | --- | --- | --- | --- | --- | --- | --- | --- | --- | --- |
|  | Univariable models | | | | |  | Multivariable models | | | |
| **a-BMI Categories (kg/m^2^)** | **N events/ patients** | **Unadjusted HR** | **95% CI** | **p-value** | **Global p-value**^†^ |  | **Adjusted HR**^†^ | **95% CI** | **p-value** | **Global p-value**^‡^ |
| <18.5 | 0/1 | NA |  |  |  |  | NA |  |  |  |
| 18.5≥ and<25 | 24/38 | Ref |  |  | 0.70 |  | Ref |  |  | 0.81 |
| 25≥ and<30 | 111/158 | 1.23 | (0.79; 1.91) | 0.36 |  |  | 1.04 | (0.66; 1.63) | 0.88 |  |
| 30≥ and <35 | 48/69 | 1.34 | (0.82; 2.19) | 0.24 |  |  | 1.16 | (0.69; 1.94) | 0.57 |  |
| ≥35 | 20/25 | 1.30 | (0.72; 2.35) | 0.39 |  |  | 1.27 | (0.68; 2.35) | 0.46 |  |
|  |  |  |  |  |  |  |  |  |  |  |
| **BMI-6mo Categories (kg/m^2^)** |  |  |  |  |  |  |  |  |  |  |
| <18.5 | 1/2 | 0.61 | (0.08; 4.49) | 0.62 | 0.73 |  | 0.51 | (0.07; 4.04) | 0.53 | 0.21 |
| 18.5≥ and<25 | 24/34 | Ref |  |  |  |  | Ref |  |  |  |
| 25≥ and<30 | 87/131 | 0.96 | (0.61; 1.51) | 0.87 |  |  | 0.66 | (0.41; 1.06) | 0.08 |  |
| 30≥ and <35 | 63/84 | 1.19 | (0.74; 1.90) | 0.48 |  |  | 0.89 | (0.54; 1.44) | 0.62 |  |
| ≥35 | 28/40 | 1.11 | (0.64; 1.92) | 0.71 |  |  | 0.99 | (0.56; 1.76) | 0.98 |  |
|  |  |  |  |  |  |  |  |  |  |  |
| **d-BMI Categories (kg/m^2^)** |  |  |  |  |  |  |  |  |  |  |
| <18.5 | 4/6 | 1.10 | (0.40; 3.03) | 0.85 | 0.14 |  | 1.19 | (0.42; 3.40) | 0.75 | <0.01 |
| 18.5≥ and<25 | 61/81 | Ref |  |  |  |  | Ref |  |  |  |
| 25≥ and<30 | 88/129 | 0.77 | (0.55; 1.07) | 0.11 |  |  | 0.59 | (0.42; 0.84) | <0.01 |  |
| 30≥ and <35 | 36/58 | 0.63 | (0.42; 0.95) | 0.03 |  |  | 0.62 | (0.41; 0.95) | 0.02 |  |
| ≥35 | 14/17 | 1.13 | (0.63; 2.03) | 0.67 |  |  | 1.19 | (0.64; 2.19) | 0.59 |  |

^†^Adjusted for sex, age at diagnosis, smoking status, treatment and year of diagnosis. The model’s baseline hazard was stratified by lymph node status, and surgery was a time dependent covariate. ^‡^Global Wald test with 4 degrees of freedom

| **Supplementary Table 7: BMI at the time of diagnosis and overall survival among EA patients in the full eligible cohort (N=406)** | | | | | | | | |
| --- | --- | --- | --- | --- | --- | --- | --- | --- |
|  | Univariable models | | | |  | Multivariable model | | |
| **d-BMI Categories (kg/m^2^)** | **N events/ patients** | **Unadjusted HR** | **95% CI** | **p-value** |  | **Adjusted HR**^†^ | **95% CI** | **p-value** |
| <18.5 | 5/7 | 1.38 | (0.56; 3.41) | 0.48 |  | 1.50 | (0.59; 3.81) | 0.40 |
| 18.5≥ and<25 | 83/111 | Ref |  |  |  | Ref |  |  |
| 25≥ and<30 | 116/177 | 0.76 | (0.57; 1.01) | 0.05 |  | 0.58 | (0.43; 0.78) | <0.01 |
| 30≥ and <35 | 45/79 | 0.59 | (0.41; 0.84) | <0.01 |  | 0.60 | (0.42; 0.88) | <0.01 |
| 35≥ | 21/32 | 0.83 | (0.52; 1.35) | 0.46 |  | 0.85 | (0.52; 1.40) | 0.52 |
|  |  |  |  | Global^‡^ P=0.04 |  |  |  | Global^‡^ P<0.01 |

One patient in the cohort (N=407) did not have d-BMI available and was not included in these models. ^†^Model was additionally adjusted for sex, age at diagnosis, smoking status, treatment and year of diagnosis. The model’s baseline hazard was stratified by clinical stage (lymph node status) at diagnosis, and surgery was coded as a time dependent covariate. ^‡^Global Wald test with 3 degrees of freedom

| **Supplementary Table 8: ΔBMI between average adult weight and weight 6 months prior to diagnosis and overall survival among EA patients (N=291)** | | | | | | | | | | | | | | | |
| --- | --- | --- | --- | --- | --- | --- | --- | --- | --- | --- | --- | --- | --- | --- | --- |
|  |  |  | | | |  |  | | | |  |  | | | |
|  |  | **Model 1** | | | |  | **Model 2** | | | |  | **Model 3** | | | |
| **∆BMI Categories (Kg/m^2^)** | N Events/ Patients | HR^†^ | 95% CI | p-value | Global p-value |  | HR^‡^ | 95% CI | p-value | Global p-value |  | HR^§^ | 95% CI | p-value | Global p-value |
| BMI loss: <0 | 57/79 | 1.06 | (0.74; 1.51) | 0.76 | 0.04^¶^ |  | 1.00 | (0.69; 1.43) | 0.99 | <0.01^¶^ |  | 1.07 | (0.75; 1.55) | 0.70 | 0.02^¶^ |
| BMI gain: ≥1.25 | 73/99 | 1.50 | (1.08; 2.10) | 0.02 |  |  | 1.75 | (1.22; 2.51) | <0.01 |  |  | 1.68 | (1.17; 2.42) | <0.01 |  |
| Stable: >0 and ≤1.25 | 73/113 | Ref |  |  |  |  | Ref |  |  |  |  | Ref |  |  |  |
| **d-BMI (kg/m^2^)** |  |  |  |  |  |  |  |  |  |  |  |  |  |  |  |
| <18.5 | 4/6 |  |  |  |  |  | 1.20 | (0.42; 3.44) | 0.74 | <0.01^#^ |  | 1.13 | (0.40; 3.23) | 0.82 | <0.01^#^ |
| 18.5≥ and<25 | 61/81 |  |  |  |  |  | Ref |  |  |  |  | Ref |  |  |  |
| 25≥ and<30 | 88/129 |  |  |  |  |  | 0.51 | (0.35; 0.73) | <0.01 |  |  | 0.54 | (0.38; 0.78) | <0.01 |  |
| 30≥ and <35 | 36/58 |  |  |  |  |  | 0.49 | (0.31; 0.77) | <0.01 |  |  | 0.54 | (0.34; 0.86) | <0.01 |  |
| ≥35 | 14/17 |  |  |  |  |  | 0.82 | (0.43; 1.57) | 0.54 |  |  | 0.94 | (0.49; 1.83) | 0.86 |  |
|  |  |  |  |  |  |  |  |  |  |  |  |  |  |  |  |
| **% bodyweight loss 6-months prior to diagnosis** |  |  |  |  |  |  |  |  |  |  |  | 1.02 | (1.00; 1.04) | 0.02 |  |

^†^Model 1: adjusted for sex, age at diagnosis, smoking status, treatment, and year of diagnosis. The model’s baseline hazard was stratified by lymph node status, and surgery was coded as a time dependent covariate. ^‡^Model 2: additionally adjusted for d-BMI. ^§^Model 3: additionally adjusted for percentage bodyweight loss 6 months before diagnosis. ^¶^Global Wald test with 2 degrees of freedom. ^#^Global Wald test with 4 degrees of freedom.

| **Supplementary Table 9: Interaction between ΔBMI and average adult BMI and overall survival among EA patients (N=291)** | | | | | | | |
| --- | --- | --- | --- | --- | --- | --- | --- |
|  |  |  | | | |  |  |
| **∆BMI Categories (Kg/m^2^)** |  | **N Events/ Patients** | **HR**^†^ | **95% CI** | **P-value** | **Interaction p-value**^‡^ |  |
| Stable: ≥0 and <1.25 | **a-BMI<27.5** | 30/55 | Ref |  |  | 0.09 |  |
| BMI loss: <0 |  | 20/23 | 1.51 | (0.83; 2.76) | 0.18 |  |  |
| BMI gain: ≥1.25 |  | 31/42 | 2.56 | (1.43; 4.59) | <0.01 |  |  |
| Stable: ≥0 and <1.25 | **a-BMI≥27.5** | 43/58 | 2.23 | (1.24; 4.00) | <0.01 |  |  |
| BMI loss: <0 |  | 37/56 | 1.57 | (0.92; 2.66) | 0.10 |  |  |
| BMI gain: ≥1.25 |  | 42/57 | 3.00 | (1.60; 5.62) | <0.01 |  |  |

^†^Adjusted for sex, age at diagnosis, smoking status, treatment, year of diagnosis, diagnosis bmi, percent body weight lost within the six months before diagnosis. The baseline hazard was stratified by lymph node status, and surgery was coded as a time dependent covariate. ^‡^Wald test p-value for interaction term.
